# The Effect of L-Carnitine, Hypotaurine, and Taurine Supplementation on the Quality of Cryopreserved Chicken Semen

**DOI:** 10.1155/2017/7279341

**Published:** 2017-04-24

**Authors:** Agnieszka Partyka, Olga Rodak, Joanna Bajzert, Joanna Kochan, Wojciech Niżański

**Affiliations:** ^1^Faculty of Veterinary Medicine, Department of Reproduction and Clinic of Farm Animals, Wroclaw University of Environmental and Life Sciences, Pl. Grunwaldzki 49, 50-366 Wroclaw, Poland; ^2^Faculty of Veterinary Medicine, Department of Immunology, Pathophysiology and Prevention Veterinary, Wroclaw University of Environmental and Life Sciences, ul. C.K. Norwida 31, 50-375 Wroclaw, Poland; ^3^Institute of Veterinary Sciences, University of Agriculture in Krakow, Al. Mickiewicza 24/28, 30-159 Krakow, Poland

## Abstract

The objective of this study was to investigate the effect of L-carnitine (LC), hypotaurine (HT), and taurine (T) on the quality of frozen-thawed chicken semen. Pooled semen samples were divided into seven aliquots (control, 1 mM LC, 5 mM LC, 1 mM HT, 10 mM HT, 1 mM T, and 10 mM T) and subjected to cryopreservation. Postthaw sperm motility was determined by IVOS system and sperm characteristics were assessed with fluorochromes and flow cytometry. The highest sperm motility and the highest percentage of viable sperm were in the HT1 group (*P* < 0.01 and *P* < 0.05) following cryopreservation. After thawing, we observed a higher percentage of sperm without apoptosis and membrane reorganization changes in the LC1 and T1 group when compared to the control (*P* < 0.05). There was a higher percentage of live sperm without lipid peroxidation (LPO) in all treatments (*P* < 0.01; *P* < 0.05), when compared to the control group. The percentage of sperm with high mitochondrial potential significantly increased with LC1, T1, and T10 (*P* < 0.05). Supplementation of the diluent with LC1, LC5, and T1 significantly (*P* < 0.05) reduced DNA susceptibility to fragmentation, compared to the control and HT1 groups. These results indicate that the addition of examined antioxidants improves the quality of cryopreserved chicken semen.

## 1. Introduction

Sperm plasma membrane possesses polyunsaturated fatty acids which are susceptible to lipid peroxidation (LPO) [1–4]. Moreover, the specific characteristics of spermatozoa such as low cytoplasm content, a large number of mitochondria, and low level of antioxidants in sperm cytoplasm make them susceptible to damage from free radicals [5, 6]. Damage of a sperm plasma membrane in the fresh semen is partially limited by the presence of antioxidant system in both spermatozoa and seminal plasma [7–9]. However, during cryopreservation, which is the sole biotechnological procedure for ex situ in vitro protection of avian genomic resource, the antioxidant protection of seminal plasma rapidly declines, as spermatozoa are extended in cryopreserving medium [10–12]. Spermatozoa are deprived of suitable protection and an increase in the intensity of LPO is observed. Therefore, artificial additives for freezing medium improving mammalian semen quality become increasingly popular in chicken reproductive techniques also.

L-Carnitine (beta-hydroxy-gamma-N-trimethylaminobutyric acid) is a water-soluble vitamin-like amino acid, which plays an important role in membrane integrity preservation, mitochondria function, and apoptosis inhibition [13]. L-Carnitine is presented in the reproductive tissue, mostly in mammalian epididymis, from where it is transported into the spermatozoa. It has been reported that sperm motility is initiated with the simultaneous increase in concentration of free L-carnitine in the epididymal lumen [14].

Hypotaurine (2-aminoethanesulfinic acid) is known to be a precursor of taurine (2-aminoethanesulfonic acid) and is the main end-product of cysteine metabolism [15]. These amino acids possess protective properties against LPO [16]. Moreover, both of them play an important role in DNA protection and reduce DNA fragmentation promoted by oxidative stress [17]. The taurine and hypotaurine are also presented in the reproductive tissues of males and females. Moreover, they play the necessary role in sperm capacitation, acrosome reaction, and fertilization [18–21].

An increasing number of reports is emphasizing the beneficial role of L-carnitine, hypotaurine, and taurine supplementation in improvement of semen quality. Several human studies have shown their positive effect on sperm motility and viability [22–26]. Moreover, animal studies revealed that the in vitro addition of these antioxidants improved sperm quality [27–31]. However, to the best of our knowledge, there are no data available about comparison of the effect of L-carnitine, hypotaurine, and taurine on sperm characteristics in cryopreserved chicken semen. Therefore, this study was conducted to investigate the in vitro effects of these three antioxidants on sperm motility, plasma membrane integrity, acrosomal damage, mitochondrial activity, the apoptotic and membrane reorganization changes, lipid peroxidation, and chromatin status in the cryopreserved chicken semen.

## 2. Materials and Methods

### 2.1. Animals and Semen Collection

Seven mature males of Green-legged Partridge breed were used in this study. Birds were kept individually in cages (70 cm × 95 cm × 85 cm) at 18–20°C, under 14L : 10D photoperiod, and fed with commercial feed for breeding flocks, without additional antioxidant supplementation. Water was provided ad libitum.

The semen was collected twice a week, by the dorsoabdominal massage method [32]. The collection was always performed by the same people and under the same conditions. All the collected ejaculates were clean and were used for study. Within 5 min after semen collection, samples from 7 individuals were pooled to obtain sufficient material for analysis. Ten semen collections were done.

### 2.2. Dilution and Cryopreservation Procedure

A EK diluent (1.4 g sodium glutamate, 0.14 g potassium citrate × H_2_O, 0.7 g glucose, 0.2 g D-fructose, 0.7 g inositol, 0.1 g polyvinylpyrrolidone, 0.02 g protamine sulfate, 0.98 g anhydrous sodium hydrogen phosphate, and 0.21 g anhydrous sodium dihydrogen phosphate were diluted to 100 mL with distilled water; pH 7.3, osmotic pressure 390 mOsmol/kg) [33] was used as the basic extender and was supplemented with the studied concentrations of antioxidants. The antioxidants L-carnitine (LC, # C0283), hypotaurine (HT, # H1284), taurine (T, # T0625), and components of the diluent were purchased from Sigma–Aldrich, St. Louis, MO, USA. Used antioxidant's doses were chosen on the basis of the previous reports [13, 22–31].

Each pooled semen sample was divided into seven aliquots (~500 *μ*L) and diluted in the ratio of 1 : 2, with the basic EK extender containing 1 mM L-carnitine (LC1), 5 mM L-carnitine (LC5), 1 mM hypotaurine (HT1), 10 mM hypotaurine (HT10), 1 mM taurine (T1), and 10 mM taurine (T10) and no antioxidant (control). The final sperm concentration of the diluted semen was about 100 × 10^7^/mL.

Semen samples were frozen in accordance with the procedure adapted from Tselutin et al. [34]. Diluted samples were cooled for 15 min at −8°C to reach 4°C. Then, cryoprotectant dimethylacetamide (DMA) was added to a final concentration of 6% and at temperature of 4°C. After five minutes of equilibration at 4°C, the solution was pipetted and plunged drop by drop (approximately 70 *μ*L per drop) directly into liquid nitrogen from a distance of about 10 cm. After three months of storage, the frozen semen was thawed in water bath at 60°C for 6 sec. For thawing, glass tubes with 3-4 pellets each were used. Ten freezing-thawing procedures were performed.

### 2.3. Assessment of Sperm Quality

#### 2.3.1. Sperm Motility Parameters

Sperm motility was evaluated using computer assisted semen analyzer (CASA) Hamilton Thorne Sperm Analyzer IVOS version 12.2l (Hamilton Thorne Biosciences, MA, USA) under 1.89 × 10 magnification. Semen was diluted in 1 : 100 (fresh) and 1 : 20 (frozen-thawed) in DMEM (Dulbecco modified medium low glucose) [35] to obtain sperm concentration of about 50 × 10^6^/mL. Three *μ*L aliquot of semen was placed in Leja4 analysis chamber (Leja, Nieuw-Vannep, Netherlands) at 35°C and evaluated. Five fields randomly selected by the computer were analyzed for each semen sample. The parameters measured were the percentage of motile sperm (MOT), the percentage of progressively motile spermatozoa (PROG), path velocity (VAP, average velocity/smoothed average position of the spermatozoa), progressive velocity (VSL, straight-line distance between the beginning and the end of the track), curvilinear line velocity (VCL, average velocity measured over the actual point-to-point track followed by the cell), straightness (STR, a measure of VCL side to side movement determined by the ratio VSL/VAP × 100), linearity (LIN, a measure of the departure of the cell track from a straight line; the ratio VSL/VCL × 100), and percentage of rapid spermatozoa (RAPID).

#### 2.3.2. Sperm Characteristics Measured by Flow Cytometry

Flow cytometric analyses were performed on a Guava EasyCyte 5 (Merck KGaA, Darmstadt, Germany) cytometer. The fluorescent probes used in the experiment were excited by an Argon ion 488 nm laser. Acquisitions were done using the GuavaSoft™ 3.1.1 software (Merck KGaA, Darmstadt, Germany). The nonsperm events were gated out based on scatter properties and not analyzed. A total of 10,000 events were analyzed for each sample.

*(1) Plasma Membrane Integrity.* Sperm membrane integrity was assessed by Live/Dead Sperm Viability Kit: SYBR-14, propidium iodide (PI) (Life Technologies Ltd., Grand Island, NY, USA), according to the protocol described by Partyka et al. [33]. The diluted samples (300 *μ*L) were stained with 5 *μ*L of SYBR-14 and 5 *μ*L of PI. Sperm SYBR-14 positive showing green fluorescence and PI negative were considered as plasma membrane intact (PMI) live sperm. 

*(2) Acrosome Integrity*. Sperm acrosome status was assessed by lectin PNA from* Arachis hypogaea* Alexa Fluor® 488 conjugate (Life Technologies Ltd., Grand Island, NY, USA). Diluted semen samples were mixed with 10 *μ*L of PNA working solution (1 *μ*g/mL) and incubated for 5 min in room temperature in the dark. After incubation, the samples were washed and 5 *μ*L of PI was added before cytometric analysis [33].

*(3) Mitochondrial Activity.* Sperm mitochondrial activity was determined using staining with the JC-1 and PI (Life Technologies Ltd., Grand Island, NY, USA). The 3 mM stock solution of JC-1 in DMSO was prepared. From each sample, 500 *μ*L of a sperm solution containing 50 × 10^6^ cell/mL was stained with 0.67 *μ*L JC-1 stock solution. The samples were incubated at 37°C in the dark for 20 min before flow cytometric analysis [36]. Sperm emitting orange fluorescence were classified as high mitochondrial membrane potential (HMMP), those emitting both green and orange fluorescence as medium, and those emitting only green fluorescence as low mitochondrial activity. 

*(4) Lipid Peroxidation.* Lipid peroxidation was evaluated using a fluorescent lipid probe C_11_-BODIPY^581/591^ (Life Technologies Ltd., Grand Island, NY, USA) as we described before [37]. One *μ*L of 2 mM C_11_-BODIPY^581/591^ in ethanol was added to diluted samples and incubated for 30 min at 37°C in the dark. Then, the samples were centrifuged at 500 ×g for 3 min and the sperm pellets were resuspended in 500 *μ*L of EK. To determine viability, the sperm was stained with PI and incubated further for 5 min at room temperature before cytometric analysis. Sperm showing only orange fluorescence (nonoxidized state of C_11_-BODIPY^581/591^) were considered live without LPO (L/LPO-).

*(5) Apoptosis and Membrane Lipid Disorder Detection. *The detected increase in sperm plasma membrane lipid packing disorder was evaluated with the stains M540 (1 mM solution in DMSO) and YO-PRO-1 (25 *μ*M solution in DMSO) (Life Technologies Ltd., Grand Island, NY, USA). Sperm were diluted after thawing to 50 × 10^6^ cell/mL with a EK diluent, and 2.7 *μ*L M540 (final concentration: 2.7 *μ*M) and 1 *μ*L of YO-PRO-1 (final concentration: 25 nM) were added to 1 mL of diluted sperm [38]. Fluorescence was measured using a FL-2 sensor, a 575 nm bandpass filter to detect M 540, and a FL-1 sensor and a 525 nm bandpass filter to detect YO-PRO-1. Cells were classified in low merocyanine fluorescence (live cells without apoptosis and membrane reorganization), high merocyanine fluorescence (live cells without apoptosis and with high membrane lipid disorder), or YO-PRO-1 positive (apoptotic sperm). 

*(6) Chromatin Status.* The acridine orange (AO, Life Technologies Ltd., Grand Island, NY, USA) stain was used to assess sperm DNA integrity as previously described [33], with minor changes. The suspension (100 *μ*L) was subjected to brief acid denaturation by mixing with 200 *μ*L of a lysis solution (Triton X-100 0.1% (v/v), NaCl 0.15 M, HCl 0.08 M, pH 1.4), held for 30 s and mixed with 600 *μ*L of AO solution (6 *μ*g AO/mL in a buffer: citric acid 0.1 M, Na_2_HPO_4_ 0.2 M, EDTA 1 mM, NaCl 0.15 M, pH 6). Samples were analyzed after 3 min of incubation. The sperm population with normal double-stranded configuration of DNA showed green fluorescence and was considered to be the main population. The cells, which showed an increased amount of red fluorescence, were located to the right of the main population indicating denatured DNA (DFI) [39].

### 2.4. Statistical Analysis

The study was replicated ten times. Significant differences between treatments and the control were determined using a one-way analysis of variance, followed by Duncan's post hoc test (*P* < 0.05) using STATISTICA (StatSoft, Inc. (2014), version 12). All percentage data were transformed to arc sin prior to analyses. The results are expressed as mean ± SD.

## 3. Results

Sperm motility parameters of chicken semen supplemented with different antioxidants are shown in Table 1. The addition of HT and T (1 and 10 mM) resulted in higher sperm motility, in comparison to the control group (*P* < 0.05, *P* < 0.01, resp.). The best result of sperm motility was achieved after 1 mM T supplementation. As shown in Table 1, no significant differences were observed between groups for other sperm motion characteristics (*P* > 0.05).

The influence of tested antioxidants on sperm parameters assessed by flow cytometry is shown in Table 2. Postthaw plasma membrane integrity was significantly (*P* < 0.05) higher in sample frozen with 1 mM T, in comparison to 10 mM HT. Moreover, in the T1 group was the highest percentage of spermatozoa with intact membrane. No significant differences were observed in sperm acrosome integrities among groups. After freezing-thawing, we observed a higher percentage of sperm without apoptosis and membrane reorganization changes in the LC1 and T1 group, when compared to the control (*P* < 0.05). There was a higher percentage of live sperm without LPO in all antioxidants treatments: LC1 (*P* < 0.05), LC5 (*P* < 0.01), HT1 (*P* < 0.05), HT10 (*P* < 0.01), T1 (*P* < 0.05), and T10 (*P* < 0.01), when compared to the control group. Percentages of sperm with high mitochondrial activity significantly increased with L-carnitine at dose 1 mM and taurine at doses 1 and 10 mM (*P* < 0.05), when compared to the control. 1 mM T produced the highest HMMP (*P* < 0.05) in relation to the control and LC5 groups. In the chromatin status assay, EK supplementation with LC1, LC5, and T1 significantly (*P* < 0.05) reduced DNA susceptibility to fragmentation, compared to the control and HT1 groups.

## 4. Discussion 

In this study, the effects of different doses of L-carnitine, hypotaurine, and taurine on sperm motility and characteristics of cryopreserved chicken sperm structures assessed by flow cytometry were investigated. Highly effective and enhanced sperm quality antioxidants are still desirable because sperm functions are altered by the freezing process and especially sperm plasma membranes are deteriorated due to membrane phase transitions.

L-Carnitine is an enhancer of lipid metabolism in animal cells. This amino acid preserves membrane integrity and mitochondria functions as well as inhibiting apoptosis. Its antioxidant properties that protect sperm membranes against toxic reactive oxygen species (ROS) are mostly related to the transfer of the products of *β*-oxidation to the mitochondria for oxidation to CO_2_ and H_2_O in the Krebs cycle [40]. Recent studies have shown that the in vitro addition of L-carnitine in spermatozoa improves their viability and motility [22, 27–29]. However, in the current study, an improvement in sperm motility was recorded when LC was not added following cryopreservation. We found that HT and T provided the greatest protective effect on motility of the frozen-thawed chicken spermatozoa, increasing it up to 10 percentage points in relation to the control. Hypotaurine and taurine are considered important for sperm motility and fertility in several species [41, 42]. The current findings relating to sperm motility improvement are in agreement with the results performed on ram [41], buffalo [42], goat [43], and fish [44]. However, there is also evidence that HT and/or T do not have an impact on sperm motility, as was observed in human [17, 24], dog [45], bull [46], and Boer goat [31]. Such discrepancies mostly depend on the species used in the investigations.

Similar observations related to the motility have been conducted in relation to sperm plasma membrane integrity. In the present study, addition of 1 mM T to the freezing medium caused improvement in the chicken sperm membrane integrity. This beneficial effect has been reported for HT and T previously by Memon et al. [31], Shiva Shankar Reddy et al. [42], and Bucak et al. [43]. L-Carnitine has also been presented as the good factor for the chicken sperm membrane stabilizer [29]. Fattah et al. [29] suggested that LC protects sperm through interaction with membrane phospholipids and modulates fluidity of plasma membrane. This occurrence helped the sperm to resist cryodamage during freezing. However, introducing HT and T to our experiment has additionally produced better results in the chicken sperm cryoviability. It seems that, in the current study, T functioned as the best agent for the chicken sperm membranes, protecting them from osmotic shock, reducing membranes' injuries, and leading to the greater resistance of spermatozoa against freeze thawing process.

As shown in Table 2, none of the tested antioxidants significantly improved chicken spermatozoal acrosome status, similarly as in the studies on mammals [28, 30, 41, 43, 46]. Previously, we obtained comparable results with other antioxidants in the chicken semen [36]. However, on the contrary, there is a report indicating that addition of HT before freezing had a positive effect on the Boer goat sperm acrosome status [31]. In the present studies, we obtained the low percentages of live and dead sperm with damaged acrosomes (about 1.5% and 4%, resp., data not presented), that could suggest that acrosome condition might be not augmented by cryopreservation or that it is not the most significant change to reduce the fertilizing ability of chicken sperm.

It is generally accepted that cryopreservation provokes loss of antioxidant defense in the semen [12, 47, 48]. Lower level of antioxidants or inhibition of antioxidant enzymes causes the oxidative stress and may damage and consequently kill the germ cells. In fact, the oxidative stress induces decrease in sperm motility, changes in membrane fluidity, and loss of plasma membrane integrity and causes DNA and protein damage [1]. Hypotaurine and taurine, as the nonenzymatic scavengers, play an important role in the inhibition of LPO and protection of the cells against the accumulation of ROS [42, 49, 50]. Moreover, LC also protects cell membranes against ROS damage, decreasing the availability of lipids for peroxidation [26]. Our study confirms the protective action of these three antioxidants against LPO. We observed a significant decrease in LPO occurrence after the use of antioxidants. The highest percentage of live sperm without LPO was observed in the samples cryopreserved with 10 mM of HT. Hypotaurine is known as a scavenger of hydroxyl radicals produced during LPO, because it is not susceptible to significant oxidation by peroxidases, preventing sperm from damage due to oxidation [51]. Thus, it can be hypothesized that the addition of these amino acids to EK extender has increased its efficacy. The intensive protective effect of these amino acids, reducing the production of ROS during the freeze thaw process, provides a proper defense against sperm lipid peroxidation. Recent studies have also revealed the positive role of LC, H, and T on LPO [29–31], showing sufficient protection against sperm damage. Shiva Shankar Reddy et al. [42] observed significantly higher antioxidant status in buffalo seminal plasma after T addition. However, there are also evidences of their ineffective role in the elimination of MDA or LPO and elevation of antioxidant activities [41, 43, 44, 46, 52].

Oxidative stress may initiate apoptosis. The most significant changes related to apoptosis are the externalization of the phosphatidylserine, DNA fragmentation, caspase activation, loss of mitochondrial membrane potential, and increase in sperm membrane permeability [53]. L-Carnitine has been shown to reduce apoptosis in different cells and organelles (cited by [40]). In fact, the antiapoptotic effect of LC has been observed in cryopreserved chicken spermatozoa [29]. Interestingly, these authors observed that, at lower concentrations, L-carnitine protected chicken sperm against apoptosis, but higher doses of 4 and 8 mM did not show such effect. Our results confirm this hypothesis. We observed a significantly higher percentage of sperm without apoptosis and membrane reorganization with 1 mM than in 5 mM LC. These LC properties are due to reduction of apoptosis through the mitochondrial pathway and are linked to downregulating the transduction of the proapoptotic Fas signal [40]. Our consecutive results from mitochondrial membrane potential could confirm this thesis. In the present study, the second effective antioxidant against apoptosis was 1 mM T. Previously, it has been reported that T administration could downregulate Fas, FasL, caspase-8, and caspase-3 protein expression in rat testes, suggesting the antiapoptotic effect of T involved in extrinsic pathway. Moreover, T showed its antiapoptotic activity through mitochondrial dependent signal [54]. However, studies are scarce, in which sperm apoptosis occurrence was assessed after T and HT supplementation; therefore, it is hard to confront our results. Only Brugnon et al. [24] reported the positive effect of HT on human sperm, but it did not confirm our findings.

As was mentioned above, the current results from sperm mitochondrial membrane potential are in agreement with results from apoptosis assessment. The highest MMP was observed in the samples cryopreserved with 1 mM LC and 1 and 10 mM T. These findings could confirm that used amino acids at presented doses protect the chicken sperm mitochondrial metabolism, through preventing electron leakage, leading to membrane potential loss, downregulation and upregulation of antiapoptotic and proapoptotic proteins, and consequently reduced oxidative damage. After induction of apoptosis, mitochondrial pores are being opened, leading to a decrease in mitochondrial membrane potential. It causes the release of proapoptotic factors into the cytoplasm, where they are activated. These factors, caspases, are central components in the apoptosis signaling cascade. It seems that, in the present experiment, LC and T played an essential role in mitochondrial ATP production [13], and antiapoptotic role was involved in intrinsic apoptotic pathway [54], as it was proposed for LC and T, respectively. Moreover, carnitine participates in mitochondria integrity maintenance, prevents free radical formation in the electron transfer chain, and inhibits activities of some ROS-generating enzymes [13]. However, Fattah et al. [29] did not observe the effect of LC on chicken sperm mitochondrial activity. Nevertheless, it should be mentioned that these authors used Rhodamine 123 for this assessment, the action of which is different from JC-1.

Apoptotic and oxidative damage also includes the loss of DNA integrity. Sperm chromatin structure is a significant determinant of sperm quality [55]. The integrity of spermatozoal DNA has vital importance for embryo development. Although sperm DNA is resistant for most of the stress because of the chromatin condensation, semen cryopreservation could cause damage to sperm DNA [56]. It has been previously found that, in chicken, freezing-thawing process caused even a twofold increase in DFI% [33]. However, in water flow, despite the high level of DNA fragmentation in the fresh semen, after cryopreservation, DFI did not increase [39]. In the present study, we found that DNA fragmentation level was significantly reduced in the semen samples, when these were cryopreserved with LC. Our findings of the protective effect of LC against DNA damage are in agreement with studies by Zhang et al. [26] and Gibb et al. [30]. In contrast, Manee-In et al. [28] and Banihani et al. [22, 23] observed no effect of LC on sperm DNA integrity. In the current study, the positive effect of 1 mM T on susceptibility of chicken sperm DNA to defragmentation was again observed. Recent studies have revealed the protective effect of T and HT against ROS attack produced by cryopreservation, reducing DNA fragmentation [24, 44]. The findings obtained could suggest that the antioxidants chosen for this experiment provided good protection of chicken sperm chromatin structure.

## 5. Conclusions

This research is the first evaluation of the influence of hypotaurine and taurine in chicken cryopreservation medium. The results of the present study demonstrate that the addition of studied antioxidants improves cryopreserved chicken semen. L-Carnitine has proven to be effective in chicken sperm protection against apoptosis, mitochondrial activity loss, and DNA defragmentation. Taurine provided the best sperm motility, viability, and mitochondrial activity and reduced sperm apoptosis and DNA damage. All antioxidants contributed to a cryoprotective effect, suppressing lipid peroxidation in the sperm membranes. Although hypotaurine provided significant increase in sperm motility and protection against LPO, taurine was found to be more effective antioxidant. Findings of this study showed that supplementation of taurine at the dose of 1 mM to semen extender was of greater benefit to the characteristic of the frozen-thawed chicken sperm and can be recommended as an additional component of the chicken freezing extender.

## Figures and Tables

**Table 1:** Motility parameters of postthawed chicken sperm (mean ± SD) supplemented with different concentrations of L-carnitine, hypotaurine, and taurine.

Groups	Motility parameters							
	MOT (%)	PROG (%)	RAPID (%)	VAP (*µ*m/s)	VSL (*µ*m/s)	VCL (*µ*m/s)	STR (%)	LIN (%)
Control	42.55 ± 12.4^Aa^	13.33 ± 6.6	22.72 ± 10.0	66.75 ± 15.7	51.42 ± 14.7	109.80 ± 17.5	70.26 ± 3.7	42.43 ± 5.7
LC1	48.06 ± 9.2^ab^	16.48 ± 4.6	27.58 ± 7.4	73.66 ± 9.2	57.45 ± 8.3	118.29 ± 10.5	70.75 ± 2.1	43.91 ± 2.9
LC5	49.94 ± 9.1^ab^	16.42 ± 6.7	25.69 ± 9.5	68.68 ± 19.5	54.27 ± 18.1	111.20 ± 22.1	71.04 ± 3.7	43.19 ± 6.3
HT1	52.18 ± 4.5^b^	17.78 ± 5.7	28.39 ± 5.8	71.78 ± 14.1	56.45 ± 14.3	114.96 ± 13.8	70.98 ± 4.0	44.17 ± 5.8
HT10	51.40 ± 5.4^b^	16.74 ± 4.6	27.64 ± 6.3	70.41 ± 12.7	54.76 ± 11.7	114.28 ± 14.1	70.42 ± 2.8	42.89 ± 4.4
T1	53.58 ± 3.6^B^	17.20 ± 5.1	28.73 ± 7.1	71.34 ± 12.2	55.23 ± 11.3	115.96 ± 14.2	70.15 ± 2.5	42.79 ± 4.1
T10	53.37 ± 4.9^B^	17.32 ± 4.6	29.16 ± 5.7	72.48 ± 14.9	56.6 ± 14.2	117.59 ± 17.8	70.79 ± 3.5	43.42 ± 5.2

Different superscripts within the same column demonstrate significant differences: ^AB^*P* < 0.01; ^ab^*P* < 0.05.

Groups: LC1: 1 mM L-carnitine; LC5: 5 mM L-carnitine; HT1: 1 mM hypotaurine; HT10: 10 mM hypotaurine; T1: 1 mM taurine; T10: 10 mM taurine. Motility parameters: MOT: motility; PROG: progressive motility; RAPID: rapid sperm motility; VAP: average path velocity; VSL: straight-line velocity; VCL: curvilinear velocity; STR: straightness, ratio VSL/VAP; LIN: linearity, ratio VSL/VCL.

**Table 2:** Mean (±SD) sperm characteristics measured by flow cytometry in frozen-thawed chicken semen supplemented with different concentrations of L-carnitine, hypotaurine, and taurine.

Groups	Spermatozoa (%)					
	PMI	L/IACR	L/APOPT-/MEMBREORG-	L/LPO-	HMMP	DFI
Control	36.60 ± 8.9^ab^	37.81 ± 4.6	39.70 ± 8.6^a^	30.57 ± 3.1^Aa^	37.48 ± 6.3^a^	2.04 ± 0.5^a^
LC1	37.60 ± 5.4^ab^	38.66 ± 8.6	44.82 ± 5.2^b^	35.13 ± 2.5^b^	42.86 ± 5.1^bc^	1.13 ± 0.6^b^
LC5	34.33 ± 4.9^ab^	38.36 ± 8.5	41.40 ± 4.4^ab^	36.58 ± 5.6^B^	39.19 ± 4.8^ac^	1.00 ± 0.6^b^
HT1	36.44 ± 8.4^ab^	37.46 ± 6.2	42.52 ± 5.3^ab^	35.24 ± 3.5^b^	41.91 ± 3.1^ab^	2.01 ± 1.1^a^
HT10	32.83 ± 6.6^a^	36.63 ± 6.5	41.49 ± 4.5^a^	38.83 ± 3.8^B^	39.55 ± 6.4^ab^	1.74 ± 1.0^ab^
T1	40.18 ± 4.4^b^	37.99 ± 5.1	45.57 ± 6.3^b^	35.06 ± 5.4^b^	44.37 ± 3.8^b^	1.21 ± 0.6^b^
T10	35.41 ± 6.5^ab^	38.78 ± 6.7	42.22 ± 4.8^ab^	36.73 ± 4.3^B^	42.69 ± 3.8^bc^	1.48 ± 0.8^ab^

Different superscripts within the same column demonstrate significant differences: ^AB^*P* < 0.01; ^ab^*P* < 0.05.

Groups: LC1: 1 mM L-carnitine; LC5: 5 mM L-carnitine; HT1: 1 mM hypotaurine; HT10: 10 mM hypotaurine; T1: 1 mM taurine; T10: 10 mM taurine. Spermatozoa: PMI: plasma membrane integrity; L/IACR: live with intact acrosome; L/APOPT-/MEMBREORG-: live without apoptosis and membrane reorganization; L/LPO-: live without LPO; HMMP: high mitochondrial membrane potential; DFI: sperm with detectable DNA fragmentation.
